# MDMA-assisted therapy for moderate to severe PTSD: a randomized, placebo-controlled phase 3 trial

**DOI:** 10.1038/s41591-023-02565-4

**Published:** 2023-09-14

**Authors:** Jennifer M. Mitchell, Marcela Ot’alora G., Bessel van der Kolk, Scott Shannon, Michael Bogenschutz, Yevgeniy Gelfand, Casey Paleos, Christopher R. Nicholas, Sylvestre Quevedo, Brooke Balliett, Scott Hamilton, Michael Mithoefer, Sarah Kleiman, Kelly Parker-Guilbert, Keren Tzarfaty, Charlotte Harrison, Alberdina de Boer, Rick Doblin, Berra Yazar-Klosinski, MAPP2 Study Collaborator Group, Charlotte Harrison, Berra Yazar-Klosinski, Wael Garas, Darrick May, Cole Marta, Susan Walker, Elizabeth Nielson, Gregory Wells, Randall Brown, Revital Amiaz, Yair Wallach, Ray Worthy, Alia Lilienstein, Amy Emerson

**Affiliations:** 1 Neuroscape, Department of Neurology, University of California, San Francisco, San Francisco, CA USA; 2 Department of Psychiatry and Behavioral Sciences, University of California, San Francisco, San Francisco, CA USA; 3 Department of Veterans Affairs, Research Service, San Francisco VA Medical Center, San Francisco, CA USA; 4 Aguazul-Bluewater, Inc, Boulder, CO USA; 5 Boston University School of Medicine, Boston, MA USA; 6 Wholeness Center, Fort Collins, CO USA; 7 Department of Psychiatry, New York University Grossman School of Medicine, New York, NY USA; 8 Zen Therapeutic Solutions, Mt. Pleasant, SC USA; 9 Nautilus Sanctuary, New York, NY USA; 10 Department of Family Medicine and Community Health, University of Wisconsin School of Medicine and Public Health, Madison, WI USA; 11 San Francisco Insight and Integration Center, San Francisco, CA USA; 12 New School Research, LLC, Los Angeles, CA USA; 13 MAPS Public Benefit Corporation, San Jose, CA USA; 14 Medical University of South Carolina, Charleston, SC USA; 15 Kleiman Consulting and Psychological Services, PC, Ivyland, PA USA; 16 KPG Psychological Services, LLC, Brunswick, ME USA; 17 University of Haifa, Haifa, Israel; 18 MAPS Israel, Hod Hasharon, Israel; 19 Tulip Medical Consulting, LLC, Port Townsend, WA USA; 20 Multidisciplinary Association for Psychedelic Studies (MAPS), San Jose, CA USA; 21 Trauma Research Foundation, Boston, MA USA; 22 Columbia University Department of Psychiatry and New York State Psychiatric Institute, New York, NY USA; 23 Chaim Sheba Medical Center, Tel Ha’Shomer, Israel; 24 Merhavim - The Medical Center for the Treatment of Mind and Soul in Beer Yaakov, Be’er Ya’akov, Israel; 25 Ray Worthy Psychiatry LLC, New Orleans, LA USA

**Keywords:** Drug development, Trauma

## Abstract

This multi-site, randomized, double-blind, confirmatory phase 3 study evaluated the efficacy and safety of 3,4-methylenedioxymethamphetamine-assisted therapy (MDMA-AT) versus placebo with identical therapy in participants with moderate to severe post-traumatic stress disorder (PTSD). Changes in Clinician-Administered PTSD Scale for DSM-5 (CAPS-5) total severity score (primary endpoint) and Sheehan Disability Scale (SDS) functional impairment score (key secondary endpoint) were assessed by blinded independent assessors. Participants were randomized to MDMA-AT (*n* = 53) or placebo with therapy (*n* = 51). Overall, 26.9% (28/104) of participants had moderate PTSD, and 73.1% (76/104) of participants had severe PTSD. Participants were ethnoracially diverse: 28 of 104 (26.9%) identified as Hispanic/Latino, and 35 of 104 (33.7%) identified as other than White. Least squares (LS) mean change in CAPS-5 score (95% confidence interval (CI)) was −23.7 (−26.94, −20.44) for MDMA-AT versus −14.8 (−18.28, −11.28) for placebo with therapy (*P* < 0.001, *d* = 0.7). LS mean change in SDS score (95% CI) was −3.3 (−4.03, −2.60) for MDMA-AT versus −2.1 (−2.89, −1.33) for placebo with therapy (*P* = 0.03, *d* = 0.4). Seven participants had a severe treatment emergent adverse event (TEAE) (MDMA-AT, *n* = 5 (9.4%); placebo with therapy, *n* = 2 (3.9%)). There were no deaths or serious TEAEs. These data suggest that MDMA-AT reduced PTSD symptoms and functional impairment in a diverse population with moderate to severe PTSD and was generally well tolerated. ClinicalTrials.gov identifier: NCT04077437.

## Abstract (web summary)

Results from the phase 3 placebo-controlled MAPP2 trial show that MDMA-assisted therapy reduces post-traumatic stress disorder (PTSD) symptoms and functional impairment in a diverse population with moderate to severe PTSD.

## Main

Post-traumatic stress disorder (PTSD) is a serious neuropsychiatric condition affecting approximately 5% of the US population each year^1^. Managing PTSD is particularly complicated in individuals experiencing the dissociative subtype of PTSD, recurrent exposure to trauma and comorbidities, such as mood disorders and alcohol and substance use disorders^2–4^. Together, these factors are associated with symptom exacerbation, treatment resistance and treatment discontinuation^3,5^. Trauma-focused psychotherapies are the gold standard treatment for PTSD. However, many individuals have persisting symptomology, and dropout rates are high^6–8^. Although the selective serotonin reuptake inhibitors (SSRIs) sertraline and paroxetine are FDA approved for treating PTSD, 35–47% of individuals do not respond to treatment^9^. More effective, therapeutic interventions are needed to address the immense individual, societal and economic burdens of PTSD^10,11^.

Mounting evidence supports substituted phenethylamine 3,4-methylenedioxymethamphetamine-assisted therapy (MDMA-AT) as a treatment for PTSD^12,13^. MDMA, an entactogen that promotes monoamine reuptake inhibition and release (primarily by inducing conformational change of pre-synaptic transporters^14–17^), effectively modulates fear memory reconsolidation, enhances fear extinction and promotes openness and prosocial behavior^18–22^. Several phase 2 trials indicated that MDMA-AT has an acceptable risk–benefit profile in individuals with PTSD^13^. A pivotal phase 3 study (MAPP1) showed that MDMA-AT was generally well tolerated and met the trial’s primary and secondary endpoints of reduced PTSD symptom severity and decreased functional impairment^12^.

Due to disparities in trauma exposure, gender-diverse and transgender individuals, ethnoracial minorities, first responders, military personnel, veterans and victims of chronic sexual abuse have a disproportionately higher risk of developing PTSD^2,23–28^. However, these diverse populations are historically underrepresented in clinical trials^29^. Here we report the results of MAPP2, the second, confirmatory phase 3 study that extends the findings of MAPP1 (refs. ^12,30^) in an ethnoracially diverse population with moderate to severe PTSD (Supplementary Table 1).

## Results

### Demographics and baseline characteristics

**Fig. 1:**
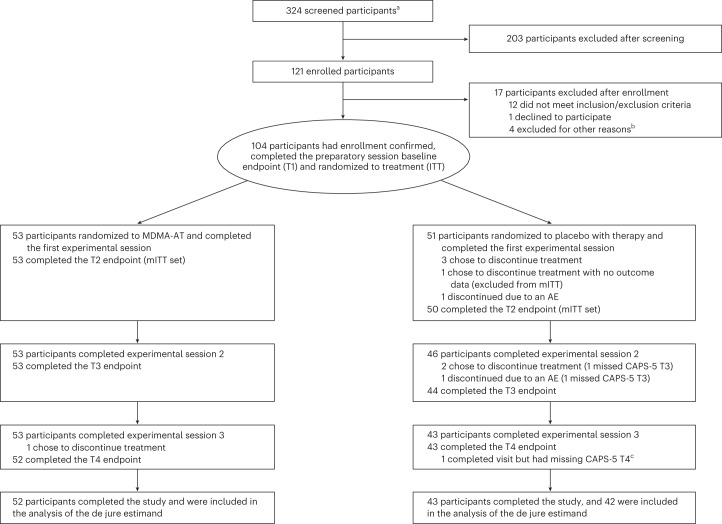
CONSORT diagram. CONSORT diagram, indicating participant numbers and disposition throughout the course of the trial. Endpoint assessments (T1, T2, T3 and T4) of CAPS-5 and SDS were conducted after each experimental session. ^a^The number of individuals after an initial phone screening who gave informed consent. ^b^Other reasons for exclusion could include withdrawal of consent, adverse event or death, discontinuation of treatment by investigator, lack of therapeutic rapport and illness or lost to follow-up. ^c^One participant in the placebo with therapy group completed the study but had missing item-level data on the final CAPS-5 assessment, and the final assessment was not included in the analysis of the de jure estimand. AE, adverse event; ITT, intention to treat; mITT, modified intention to treat; T, time of endpoint assessment; T1, baseline; T2, after experimental session 1; T3, after experimental session 2; T4, 6–8 weeks after experimental session 3 (18 weeks after baseline).

Participants were recruited from 21 August 2020 to 18 May 2022 (last participant visit on 2 November 2022). Overall, 324 individuals were screened, and 121 were enrolled. Of these, 17 individuals did not meet enrollment confirmation after initiation of preparation therapy, and 104 were confirmed for randomization: 53 were assigned to MDMA-AT and 51 to placebo with therapy (Fig. 1). Ninety-four participants completed the study, and nine discontinued (*n* = 1 MDMA-AT; *n* = 8 placebo with therapy) (Fig. 1 and Supplementary Table 3).

Baseline characteristics were generally similar between groups (Table 1). In total, 74 of 104 (71.2%) participants were assigned female sex at birth, with a higher proportion in the placebo with therapy group (42/51, 82.4%) than the MDMA-AT group (32/53, 60.4%). Participants were ethnically and racially diverse: 35 of 104 (33.7%) participants identified their race as other than White, and 28 of 104 (26.9%) identified their ethnicity as Hispanic/Latino. The mean (s.d.) duration of PTSD was 16.2 (13.3) years. The mean (s.d.) Clinician-Administered PTSD Scale for DSM-5 (CAPS-5) score at baseline was 39.0 (6.6) and was similar between groups. Overall, 28 of 104 (26.9%) and 76 of 104 (73.1%) participants had moderate and severe PTSD, respectively; the dissociative subtype was present in 24 of 104 (23.1%) participants.

**Table 1:** Demographics and clinical characteristics of participants at baseline

Characteristic	MDMA-AT (*n* = 53)	Placebo with therapy (*n* = 51)
Age (years), mean (s.d.)	38.2 (11.0)	40.0 (9.6)
Sex assigned at birth, *n* (%)		
Male	21 (39.6)	9 (17.6)
Female	32 (60.4)	42 (82.4)
Ethnicity, *n* (%)		
Hispanic or Latino	17 (32.1)	11 (21.6)
Not Hispanic or Latino	36 (67.9)	39 (76.5)
Race, *n* (%)		
American Indian/Alaska Native	0	2 (3.9)
Asian	5 (9.4)	6 (11.8)
Black or African American	5 (9.4)	3 (5.9)
Native Hawaiian/Pacific Islander	0	1 (2.0)
White	37 (69.8)	32 (62.7)
Multiple	6 (11.3)	7 (13.7)
BMI (kg m^−2^), mean (s.d.)	26.3 (5.6)	24.7 (4.9)
Duration of PTSD (years), mean (s.d.)	16.3 (14.3)	16.1 (12.4)
Dissociative subtype of PTSD, *n* (%)	13 (24.5)	11 (21.6)
Psychiatric disorder, *n* (%)		
Comorbid major depression	49 (92.5)	51 (100)
Suicidal ideation	44 (83.0)	47 (92.2)
Trauma history, *n* (%)		
Developmental trauma events	49 (92.5)	43 (84.3)
Combat exposure	9 (17.0)	6 (11.8)
Veteran status	9 (17.0)	7 (13.7)
Multiple trauma events	40 (75.5)	45 (88.2)
Pre-study PTSD medication, *n* (%)^a^		
Paroxetine	1 (1.9)	1 (2.0)
Sertraline	15 (28.3)	10 (19.6)
Pre-study therapy, *n* (%)		
Cognitive behavioral therapy	15 (28.3)	14 (27.5)
Cognitive processing therapy	1 (1.9)	1 (2.0)
Dialectical behavioral therapy	4 (7.5)	2 (3.9)
Eye movement desensitization reprocessing	17 (32.1)	18 (35.3)
Group therapy	9 (17.0)	15 (29.4)
Holotropic breathwork	0	3 (5.9)
Prolonged exposure therapy	2 (3.8)	0
Psychodynamic therapy	15 (28.3)	11 (21.6)
Other	41 (77.4)	42 (82.4)
Baseline CAPS-5 total severity score, mean (s.d.)	39.4 (6.6)	38.7 (6.7)
Baseline PTSD severity, *n* (%)		
Moderate (CAPS-5 score 28–34)	13 (24.5)	15 (29.4)
Severe (CAPS-5 score ≥35)	40 (75.5)	36 (70.6)
Baseline C-SSRS score, mean (s.d.)		
Suicidal ideation	0.4 (0.8)	0.3 (0.6)
Ideation intensity	3.0 (5.5)	2.8 (5.3)
Baseline BDI-II total score, mean (s.d.)	25.4 (11.9)	25.5 (11.3)
ACE questionnaire score, mean (s.d.)	4.8 (2.9)	4.5 (2.7)
Prior report of MDMA use, *n* (%)		
Lifetime reported use	22 (41.5)	26 (51.0)
Reported use in the past 10 years	13 (24.5)	18 (35.3)

^a^Medications were tapered and washed out before baseline assessments and the first experimental session, in accordance with the protocol. ACE, adverse childhood experience; BMI, body mass index.

### Primary outcomes

**Fig. 2:**
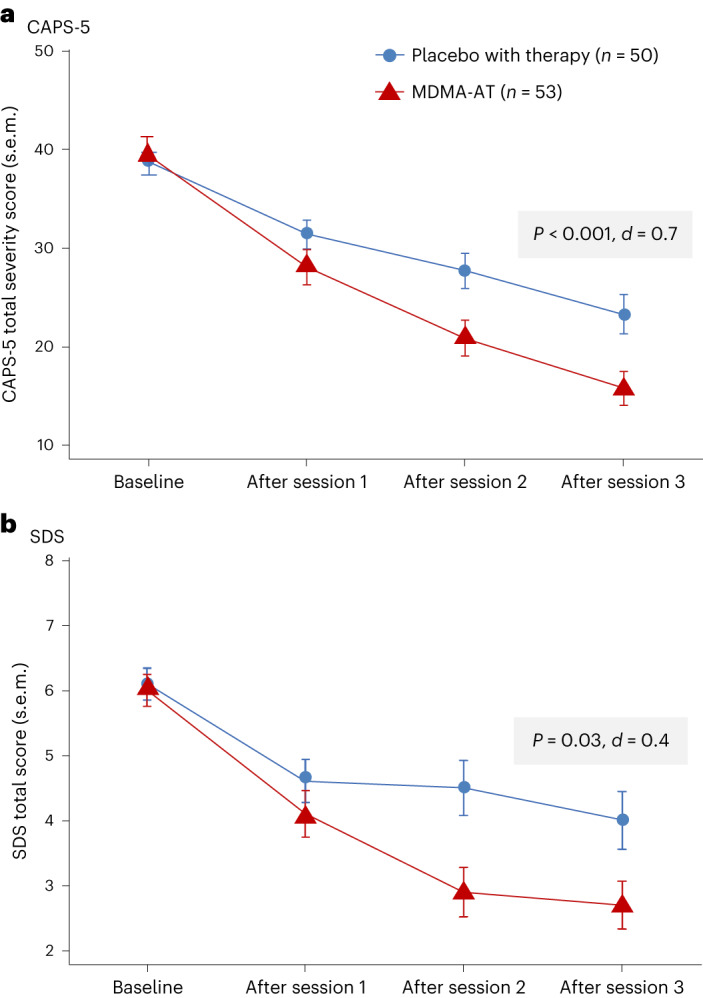
Measures of efficacy in the MDMA-AT and placebo with therapy groups. **a**, LS mean change (±s.e.m.) in CAPS-5 total severity score from baseline to after session 3 (primary outcome) for placebo with therapy (*n* = 50) versus MDMA-AT (*n* = 53, *P* < 0.001, Cohen’s *d* = 0.7). **b**, LS mean change (±s.e.m.) in SDS total score from baseline to after session 3 (key secondary outcome) for placebo with therapy (*n* = 50) versus MDMA-AT (*n* = 53, *P* = 0.03, Cohen’s *d* = 0.4). Source data

MDMA-AT significantly attenuated PTSD symptomology versus placebo with therapy, as measured by a reduction in CAPS-5 total severity score from baseline to 18 weeks. Mixed models for repeated measures (MMRM) analysis of the de jure estimand showed a least squares (LS) mean (95% confidence interval (CI)) change of −23.7 (−26.94, −20.44) for MDMA-AT versus −14.8 (−18.28, −11.28) for placebo with therapy (treatment difference: −8.9 (−13.70, −4.12), *P* < 0.001; Fig. 2a). The Cohen’s *d* effect size of MDMA-AT versus placebo with therapy was *d* = 0.7; the within-group effect sizes were *d* = 1.95 for MDMA-AT and *d* = 1.25 for placebo with therapy. MMRM analysis of the de facto estimand revealed an LS mean change (95% CI) in CAPS-5 scores of −23.7 (−26.97, −20.47) for the MDMA-AT group versus −14.8 (−18.24, −11.33) for the placebo with therapy group (*P* < 0.001).

### Secondary outcomes

MDMA-AT significantly mitigated clinician-rated functional impairment, as measured by a reduction in the Sheehan Disability Scale (SDS) from baseline. MMRM analysis of the de jure estimand revealed that the LS mean change (95% CI) in SDS total scores was −3.3 (−4.03, −2.60) with MDMA-AT versus −2.1 (−2.89, −1.33) with placebo with therapy (treatment difference: −1.20 (−2.26, −0.14); *P* = 0.03, *d* = 0.4; Fig. 2b). Improvements were observed across all domains, including family life, social life and work life (Supplementary Table 4).

### Exploratory outcomes

**Fig. 3:**
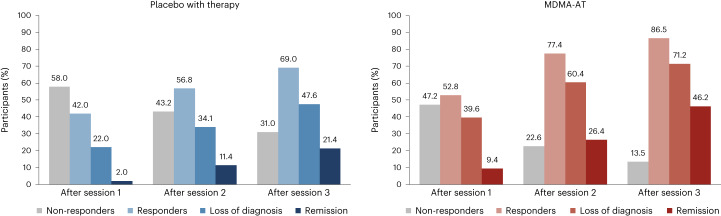
Treatment response and remission in the MDMA-AT (*n* = 53) and placebo with therapy (*n* = 50) groups. A ≥10-point reduction in CAPS-5 total severity score was considered to be clinically meaningful. Responders (≥10-point reduction from baseline), loss of diagnosis (≥10-point reduction from baseline and no longer meeting PTSD diagnostic criteria) and remission (loss of diagnosis and CAPS-5 total severity score of 11 or less) were tracked in both groups as a percentage of participants. Non-responders were defined as any CAPS-5 total severity score change <10-point reduction from baseline. Source data

In the MDMA-AT group, 45 of 52 (86.5%) participants were responders with a clinically meaningful improvement at 18 weeks after baseline, defined as a ≥10-point reduction in CAPS-5 total severity score, versus 29 of 42 (69.0%) in the placebo with therapy group (Fig. 3). By study end, 37 of 52 (71.2%) participants in the MDMA-AT group no longer met DSM-5 criteria for PTSD versus 20 of 42 (47.6%) participants in the placebo with therapy group. Furthermore, 24 of 52 (46.2%) participants in the MDMA-AT group and nine of 42 (21.4%) participants in the placebo with therapy group met remission criteria (Fig. 3). The net number of participants needed to treat for each responder analysis group was as follows: responder, six; non-responder, six; loss of diagnosis, four; remission, four.

Covariate analyses demonstrated similar responses to treatment regardless of disease severity, risk of hazardous alcohol or substance use disorder, severe adverse childhood experiences or dissociative subtype PTSD. The only measured exploratory covariate with a significant interaction with treatment was lifetime history of SSRI use, which was associated with improved efficacy of MDMA-AT (*P* = 0.02; Supplementary Table 5). Covariates significantly impacting the main effect were sex assigned at birth and baseline Beck Depression Inventory (BDI)-II score; female sex assigned at birth and baseline BDI-II score ≥23 were both associated with improved outcomes irrespective of treatment assignment (*P* < 0.05).

A blinding survey conducted at study termination showed that 33 of 44 (75.0%) participants in the placebo with therapy group were certain or thought they received placebo, whereas nine of 44 (20.5%) participants inaccurately thought that they received MDMA, and two of 44 (4.5%) participants could not tell. In the MDMA-AT group, 49 of 52 (94.2%) participants were certain or thought that they received MDMA; one of 52 (1.9%) participants inaccurately thought that they received placebo; and two of 52 (3.8%) participants could not tell (Supplementary Table 6). When asked for the reason for their belief in treatment assignment, most participants in the MDMA-AT group reported attributing their response on the blinding survey to experiencing positive mental or emotional effect (45/52 (86.5%)) and positive physical effect (29/52 (55.8%)), whereas most of the participants in the placebo with therapy group reported experiencing no effect (28/44 (63.6%)).

### Safety

Most participants (102/104, 98.1%) experienced at least one treatment-emergent adverse event (TEAE) during the study (Table 2); seven experienced a severe TEAE (MDMA-AT, *n* = 5 (9.4%); placebo with therapy, *n* = 2 (3.9%)). None had a serious TEAE. Two participants (3.9%) in the placebo with therapy group discontinued treatment due to TEAEs. Frequently reported TEAEs (occurring with incidence >10% and at least twice the prevalence in the MDMA-AT group versus the placebo with therapy group) included muscle tightness, nausea, decreased appetite and hyperhidrosis (Table 2). These were mostly transient and of mild or moderate severity. At least one treatment-emergent adverse event of special interest (TEAESI) occurred in six of 53 (11.3%) participants in the MDMA-AT group and three of 51 (5.9%) participants in the placebo with therapy group (Table 2). No TEAESIs of MDMA abuse, misuse, physical dependence or diversion were reported.

**Table 2:** Adverse events occurring during treatment

	MDMA-AT (*n* = 53)	Placebo with therapy (*n* = 51)
Summary of TEAEs and TEAESIs, *n* (%)		
Participants with ≥1 TEAE	53 (100)	49 (96.1)
Participants with ≥1 severe TEAE	5 (9.4)	2 (3.9)
Participants with ≥1 serious TEAE	0	0
Participants with ≥1 TEAE leading to study discontinuation	0	2 (3.9)
Participants with ≥1 TEAESI	6 (11.3)	3 (5.9)
Most common^a^ TEAEs, *n* (%)		
Muscle tightness	31 (58.5)	13 (25.5)
Nausea	24 (45.3)	11 (21.6)
Decreased appetite	19 (35.8)	5 (9.8)
Hyperhidrosis	18 (34.0)	3 (5.9)
Feeling hot	14 (26.4)	6 (11.8)
Feeling cold	11 (20.8)	3 (5.9)
Paresthesia	10 (18.9)	1 (2.0)
Chest discomfort	9 (17.0)	2 (3.9)
Dry mouth	9 (17.0)	4 (7.8)
Chills	8 (15.1)	1 (2.0)
Feeling jittery	8 (15.1)	0
Restlessness	8 (15.1)	2 (3.9)
Vision blurred	8 (15.1)	0
Bruxism	7 (13.2)	1 (2.0)
Nystagmus	7 (13.2)	1 (2.0)
Mydriasis	6 (11.3)	0
Tremor	6 (11.3)	0

^a^The most common TEAEs occurring with incidence >10% and at least twice the prevalence in the MDMA-AT group versus the placebo with therapy group.

Eight participants (MDMA-AT, *n* = 7; placebo with therapy, *n* = 1) experienced cardiac TEAEs, which included palpitations (MDMA-AT, *n* = 5 (9.4%); placebo with therapy, *n* = 1 (2.0%)) and tachycardia (MDMA-AT, *n* = 2 (3.8%)); all were mild. Nine participants (MDMA-AT, *n* = 7; placebo with therapy, *n* = 2) experienced vascular TEAEs; all were mild, except for one participant in the MDMA-AT group who had a history of hypertension, who was not taking anti-hypertensive medications and who experienced a TEAE of moderate hypertension (Supplementary Table 7). Five participants had cardiac TEAESIs: four participants in the MDMA-AT group and one participant in the placebo with therapy group reported palpitations (Supplementary Table 7). Participants in the MDMA-AT group experienced temporary dose-dependent increases in mean blood pressure (BP) and pulse during experimental sessions compared to the placebo with therapy group (Supplementary Table 8).

Transient increases in heart rate and BP were expected and were observed during experimental sessions in a dose-dependent manner. Greater fluctuations in BP were seen during experimental sessions 2 and 3 in the participants treated with MDMA, most likely due to the higher doses of MDMA administered. These transient elevations did not require clinical intervention, including among the subset of participants with well-controlled hypertension. Because the current dosing regimen involves administering a single, split drug dose under observation, for a limited number of times, each after a lengthy washout, cardiovascular risk is likely to have been sufficiently mitigated by the study procedures and screening measures.

Psychiatric TEAEs occurred at a similarly high frequency in both groups (MDMA-AT, *n* = 44 (83.0%); placebo with therapy, *n* = 37 (72.5%)), with suicidal ideation, insomnia and anxiety reported most frequently. Psychiatric TEAEs were mostly mild to moderate; three severe events occurred in the MDMA-AT group (5.7%; *n* = 1 each: dissociation, flashback and grief reaction) and two in the placebo with therapy group (3.9%; *n* = 1 each: agitation and anxiety). No severe TEAEs of suicidal ideation or behavior were reported. Two participants in the MDMA-AT group had suicidality TEAESIs of suicidal ideation, one of whom engaged in non-suicidal self-injurious behavior. Two participants in the placebo with therapy group had suicidality TEAESIs; one engaged in non-suicidal self-injurious behavior, and one had suicidal ideation and trichotillomania (Supplementary Table 9).

More than 80% (87/104) of participants had a lifetime history of suicidal ideation; 13 of 53 (24.5%) in the MDMA-AT group and 12 of 51 (23.5%) in the placebo with therapy group reported suicidal ideation during the final preparation session (V4). The number of participants reporting positive suicidal ideation varied throughout the study but collectively never exceeded baseline values in either group (Supplementary Fig. 2). Three participants (two MDMA-AT and one placebo with therapy) had treatment-emergent active suicidal ideation with at least some intent to act (Columbia-Suicide Severity Rating Scale (C-SSRS) score of 4 or 5), which was observed on five occasions (MDMA-AT, three events; placebo with therapy, two events) (Supplementary Fig. 2). Of these, one participant in the MDMA-AT group with no suicidal ideation at baseline had the emergence of active suicidal ideation with at least some intent to act.

## Discussion

In this confirmatory phase 3 study of participants with moderate to severe PTSD, MDMA-AT significantly improved PTSD symptoms and functional impairment, as assessed by CAPS-5 and SDS, respectively, compared to placebo with therapy over 18 weeks. Notably, 45 of 52 (86.5%) participants treated with MDMA-AT achieved a clinically meaningful benefit, and 37 of 52 (71.2%) participants no longer met criteria for PTSD by study end. In a historic first, to our knowledge, for psychedelic treatment studies, participants who identified as ethnically or racially diverse encompassed approximately half of the study sample. These findings confirm and extend the results observed in MAPP1 (ref. ^12^), with general consistency across endpoints.

Given the diverse population and degree of participant complexity, the replication of efficacy is particularly notable. In our study, 26.9% (28/104) of participants expressed moderate PTSD, whereas, in MAPP1, all participants expressed severe PTSD^12^. A substantial proportion of participants displayed comorbid features associated with high treatment resistance^5^, such as major depression, multiple sources of trauma (including childhood and combat trauma) and dissociative subtype PTSD. In keeping with MAPP1, treatment was not significantly affected by disease severity, risk of hazardous alcohol or substance use disorder, severe adverse childhood experiences or dissociative subtype. Furthermore, there was no observed site-to-site variability and no differential effect if participants stayed overnight after the experimental session. However, lifetime history of SSRIs, female sex assigned at birth and BDI-II score ≥23 at baseline were associated with positive impacts on outcomes and may warrant further study based on the exploratory nature of these analyses.

MDMA simultaneously induces prosocial feelings and softens responses to emotionally challenging and fearful stimuli^19^, potentially enhancing the ability of individuals with PTSD to benefit from psychotherapy by reducing sensations of fear, threat and negative emotionality^18,19^. The low dropout rate for MDMA-AT has been replicated across seven studies, suggesting that MDMA induces a true shift in participant engagement^12,13^. In contrast, a recent study comparing psychotherapies in veterans with PTSD reported dropout rates of 55.8% and 46.6% for prolonged exposure and cognitive processing therapy, respectively^31^. The MAPP2 dropout rate was 1.9% (1/53) in the MDMA-AT group and 15.7% (8/51) in the placebo with therapy group. The higher proportion of dropouts in the placebo with therapy group relative to MDMA-AT could be attributed to participants receiving less effective treatment and to disappointment from ineffective therapeutic blinding, although blinding survey data showed that not all participants correctly identified the treatment that they received.

Consistent with MAPP1, no new major safety issues were reported. Common TEAEs were similar to previous studies and consistent with expected effects of MDMA^12,32^. Rates of cardiac TEAEs were low, and increases in BP and pulse were mild, transient and consistent with MDMA’s sympathomimetic effects^18,33,34^. Consistent with PTSD, suicidal ideation was observed in both groups. MDMA did not appear to increase this risk, and no suicidal behavior was observed. C-SSRS scores varied throughout the study but never exceeded baseline values for either group. Notably, there were five total events of treatment-emergent C-SSRS scores of 4 or 5: three in the MDMA-AT group and two in the placebo with therapy group. MAPP2 enrolled participants with a history of suicidality but excluded those with a current, serious imminent suicide risk; thus, special attention to this vulnerable population is warranted in future studies. In alignment with MAPP1 (ref. ^12^), there were no reports of problematic MDMA abuse or dependence, including in participants with histories of, or current, alcohol and substance use disorders. However, it is important to note that participants with any substance use disorder other than cannabis or alcohol in the 12 months before enrollment were excluded from MAPP2, as were participants with severe or moderate (in early remission) alcohol or cannabis use disorder. However, exploratory findings from the MAPP1 phase 3 trial indicated that MDMA-AT was actually associated with a significantly greater reduction in mean Alcohol Use Disorder Identification Test change scores compared to placebo with therapy, suggesting that the effects of MDMA-AT on hazardous alcohol use secondary to PTSD should be further studied^35^. Long-term data are also needed to assess the risk of MDMA abuse or misuse after study participation.

Although the sample sizes of the MAPP1 and MAPP2 phase 3 studies had 90% statistical power and were developed with guidance from the FDA to ensure adequate, rigorous testing of outcomes, these evaluations did not extend further than 2 months after therapy and were intended to support an acute treatment course. To support these studies, data from the ongoing follow-up of participants from phase 2 and 3 studies (ClinicalTrials.gov Identifier: NCT05066282) will be important for further assessment of the long-term effectiveness of MDMA-AT in participants with PTSD. It is of interest to note that pooled phase 2 analyses of participants with at least 12 months of follow-up after their final MDMA-AT session have shown that LS mean CAPS-IV scores continue to improve between the final session and follow-up^32^.

Several limitations may impact the integration of MDMA-AT into clinical care, including the exclusion of participants with high suicide risk, comorbid personality disorders and underlying cardiovascular disease. Observed effect sizes for MDMA-AT (between-group, *d* = 0.7; within-subject, *d* = 1.95) were similar to MAPP1 (ref. ^12^) (between-group, *d* = 0.91; within-subject, *d* = 2.1), and, although higher than those observed in SSRI studies (ranging from 0.09 to 0.56 versus placebo for sertraline and paroxetine^36^), the superiority of MDMA-AT over SSRIs cannot be assumed without a direct comparison. The complex relationship between SSRI use/history and MDMA-AT treatment efficacy was beyond the scope of the current statistical analysis plan and sample size but will be important to consider in future studies. In addition, further study of MDMA with other forms of psychotherapy for PTSD should be explored.

The notable effect seen in the placebo with therapy arm could suggest the standalone value of the manualized inner-directed therapy that was developed for use with MDMA. Additional head-to-head studies will need to be conducted to evaluate whether this form of manualized therapy provides greater value in the treatment of PTSD than the current first-line cognitive behavioral therapy and prolonged exposure therapy treatments^37^.

Although treatment expectancy, per se, was not measured in this study, prospective treatment expectancy would likely have been high in both study arms, with random assignment expected to distribute this equally between groups. Although expectancy effects are a well-known issue in psychiatric clinical trials and are intertwined with the observation of treatment benefit during a trial^38^, several observations support expectancy mitigation in the current study: (1) the groups did not separate after the first experimental session; (2) placebo with therapy dropouts did not uniformly occur after the first experimental session; and (3) blinding survey data (Supplementary Table 6) showed that not all participants correctly identified the treatment that they received.

The therapists who participated in this study were required to complete the sponsor’s training program (see Supplementary Methods for further details). To ensure consistent clinical practice and to mitigate harm, it may be of benefit for prescribers to complete additional training and continuing education if MDMA-AT is approved for use by a regulatory agency.

This confirmatory phase 3 trial showed consistent benefits of MDMA-AT in an ethnoracially diverse group of individuals with longstanding moderate to severe PTSD and numerous comorbidities. The dropout rate was low, and treatment was generally well tolerated. These findings represent the culmination of over two decades of research^39^, and, together with MAPP1, indicate that further consideration of this treatment in individuals with moderate to severe PTSD is warranted.

## Methods

### Study design and oversight

This multi-site, randomized, double-blind, placebo-controlled study assessed the efficacy and safety of MDMA-AT versus placebo with therapy in participants diagnosed with moderate or severe PTSD (NCT04077437). Thirteen study sites (11 in the United States and two in Israel, both institutional and private) participated. The trial was conducted in accordance with the Good Clinical Practice guidelines of the International Council for Harmonization and with the ethical principles of the Declaration of Helsinki. An independent data monitoring committee ensured that the study was conducted safely and had sufficient sample size. The review boards and institutions that approved the study protocol are listed in the Supplementary Methods.

### Participants

After written informed consent, participants were screened for eligibility. Adults (≥18 years of age) meeting the full DSM-5 criteria for current PTSD per CAPS-5 assessment^40,41^ and a CAPS-5 total severity score ≥28 (moderate or higher severity) with symptom duration of ≥6 months were eligible for enrollment confirmation. During the Preparation Period that preceded the Treatment Period, participants were tapered off all psychiatric medications before baseline to avoid potential drug interactions and confounding efficacy (Supplementary Fig. 1). Full inclusion and exclusion criteria are outlined in the Supplementary Methods.

### Randomization and masking

Participants were randomized in a 1:1 allocation and in a blinded fashion to the MDMA-AT and placebo with therapy groups, stratified by clinical site. Randomization was managed via an interactive web randomization system (IWRS) (IT Clinical version 11.0.1) based on a centralized randomization schedule developed by an independent third-party vendor to maintain blinding.

A central pool of blinded independent assessors was used to mitigate the risk of functional unblinding^42^. Assessors were trained and supervised by independent consultants with expertise in PTSD diagnostics and the CAPS-5 to ensure inter-rater reliability and validity of assessments. Supervision involved reviewing each assessor’s first two assessments as well as 20% of all assessments (chosen at random) throughout the study, with each review resulting in detailed feedback for the assessor. The independent assessors were blinded to the general study design, study visit, treatment assignment, number of treatments received and any safety data for the participant. Participants were instructed to withhold their opinion on treatment group assignment and the number of completed visits from the independent assessors. Each assessor conducted no more than one CAPS-5 assessment with each participant to reduce potential bias and expectancy effect from having conducted repeat CAPS-5s with a participant. Assessors were also vetted before their onboarding to ensure that there were no conflicts of interest (such as other involvement within the Multidisciplinary Association for Psychedelic Studies (MAPS) organization or a bias toward MDMA-AT), and assessors were instructed to not expose themselves to scholarly presentations and papers related to MDMA-AT for PTSD to maintain their blinding to study design.

To ensure that all site and sponsor staff were shielded from study outcomes, the blinded independent assessor pool collected and stored outcome measures in a dedicated database that was separate from the blinded clinical database. A blinding survey was conducted at study termination (visit 20) to assess if participants thought that they received MDMA or placebo.

### Procedures

Trial procedures were consistent with MAPP1 (ref. ^12^). Enrolled participants underwent three 90-min preparation sessions with a two-person therapy team, including at least one licensed therapist, and were then randomized 1:1 to receive MDMA-AT or placebo with therapy for approximately 3 months. The treatment period consisted of three 8-h dosing sessions, in conjunction with therapy, spaced approximately 1 month apart. Therapy was conducted by trained personnel in accordance with the MAPS MDMA-AT treatment manual (https://maps.org/treatment-manual) and trial protocol. During experimental sessions, and in keeping with the dosing in MAPP1, participants received a split dose of 120–180 mg of MDMA or placebo. For the first experimental session, the initial dose of 80 mg was followed by a supplemental half-dose of 40 mg. In the second and third experimental sessions, the initial dose of 120 mg was followed by a supplemental half-dose of 60 mg. The supplemental half dose was administered 1.5–2 h after the initial dose. Participants in both treatment groups received identical therapy. The 120-mg (80 mg + 40 mg) split dose was selected for the first experimental session in phase 3 trials to allow patients to acclimate to the treatment regimen using a clinical titration approach based on clinician recommendations from a phase 2 trial in veterans and first responders^13^. During the second and third experimental sessions, doses were escalated to 180 mg (120 mg + 60 mg), as this was the most frequently studied efficacious dose in phase 2 trials. This dosing regimen also provides clinicians with the option of dose adjustments if needed.

Within the MDMA-AT group, three participants did not undergo dose escalation in experimental sessions 2 and 3, and two participants experienced dose administration timing errors (Supplementary Table 2). Each experimental session was followed by three 90-min integration sessions to support participants in processing and understanding their experience (Supplementary Fig. 1). Full procedures, including details on therapy teams and training, are outlined in the Supplementary Methods.

### Outcomes

Independent assessors conducted CAPS-5 and SDS outcome assessments at baseline, after experimental sessions 1 and 2 and 6–8 weeks after experimental session 3 (18 weeks after baseline) via video interviews. Primary and secondary objectives were mean change in CAPS-5 total severity and SDS scores, respectively, for MDMA-AT versus placebo with therapy from baseline to 18 weeks after baseline.

Exploratory outcome measurements included characterization of the treatment response and differences between the treatment groups by demographics and characteristics. Responder analyses were based on categorical diagnostic assessment data and the CAPS-5 total severity score assessment. PTSD severity was defined using the CAPS-5 total severity score as follows: asymptomatic (0–10), mild (11–22), moderate (23–34), severe (35–46) and extreme (47+) (ref. ^41^). A ≥10-point reduction in CAPS-5 total severity score was considered to be clinically meaningful as agreed upon with the FDA through a Special Protocol Assessment. Four responder categories were derived and compared at each post-experimental session visit using CAPS-5 scores. These categories were: non-responder (<10-point reduction from baseline), responder (≥10-point reduction from baseline), loss of diagnosis (≥10-point reduction from baseline and no longer meeting PTSD diagnostic criteria) and remission (CAPS-5 total severity score of 11 or less and no longer meeting PTSD diagnostic criteria).

Safety objectives included assessment of differences between groups in severity, incidence and frequency of TEAEs, serious TEAEs, TEAESIs, suicidal ideation and behavior and vital signs. TEAEs were defined as any adverse event that occurred during the treatment period from the first experimental session to the last integration session. The severity of TEAEs was determined by the site physician as mild (no limitation in normal daily activity), moderate (some limitation in normal daily activity) or severe (unable to perform normal daily activity). A serious TEAE was defined as any unforeseen medical event at any dose of the drug that resulted in death; was life-threatening; required inpatient hospitalization; caused significant disability or incapacity; resulted in a congenital anomaly or birth defect; or required intervention to prevent permanent impairment or damage. Serious TEAEs also included any event, based on medical judgement, that jeopardized the participant or may have required intervention to prevent one of the events listed previously. With the exception of serious adverse event reporting, relatedness to study drug was not assessed by investigators, to preserve blinding. In an effort to identify common adverse events that may be most related to MDMA, TEAEs occurring with incidence >10% and at least twice the prevalence in the MDMA-AT group versus the placebo with therapy group are reported. Suicidality was tracked at each study visit using the C-SSRS (see the Supplementary Methods for more information).

### Statistical analysis

SAS version 9.4 (SAS Institute) was used for analyses. Sample size was calculated to achieve a power of 90% at an alpha of 0.0499.

Efficacy was tested using an MMRM analysis comparing the change from baseline to 18 weeks after baseline in CAPS-5 and SDS scores between treatment groups in two-sided tests with alpha set at 0.0499. The alpha was adjusted to account for an administrative interim analysis for sample size re-estimation conducted after all participants were enrolled and 60% of primary endpoint data had been collected. Fixed effects were treatment, visit, treatment group by visit interaction and dissociative subtype; baseline CAPS-5 score was a covariate. Primary and secondary efficacy analyses used a de jure (related to initially randomized treatment) estimand and a supportive de facto (treatment policy) estimand of the modified intention-to-treat population, which required exposure to MDMA or placebo and at least one follow-up CAPS-5 assessment, as in MAPP1 (ref. ^12^). The de jure dataset included all available data, except for 12 (one MDMA-AT and 11 placebo with therapy) outcome measurements taken after treatment discontinuation in analysis of treatment efficacy (Supplementary Table 3). Missed observations were considered missing at random (MAR), and choice of this assumption was tested with a tipping point analysis (Supplementary Methods).

In additional exploratory analyses, 13 covariates were assessed in the model, with alpha set at 0.0499: age, sex (self-reported), prior use of selective SSRIs, work disability, disease severity, PTSD duration, dissociative subtype, overnight site stay, site ID, moderate depression (as measured by the BDI-II), severe adverse childhood experiences and moderate alcohol and substance use disorder risk (as measured by the Drug Use Disorders Identification Test and the Alcohol Use Disorders Identification Test). Analyses of primary or secondary outcomes by gender were not planned a priori; some exploratory analyses included sex as a covariate (Supplementary Methods).

Safety analysis evaluated TEAEs at the participant level, including all participants who received MDMA or placebo. Causal association with MDMA was determined based on relative incidence of TEAEs with at least a two-fold difference between groups.

### Adverse events of special interest

In accordance with FDA guidance, special attention was paid to a subset of adverse events, TEAESIs, relating to cardiac function, suicide risk and MDMA abuse, misuse or diversion. TEAESIs involving cardiac function that could be indicative of QT prolongation or cardiac arrhythmias were collected, including torsade de pointes, sudden death, ventricular extrasystoles, ventricular tachycardia, ventricular fibrillation and flutter, non-postural syncope and seizures. TEAESIs involving suicide risk included suicide, suicide attempts, self-harm associated with suicidal ideation, suicide ideation assessed as a score of 4 or 5 on the C-SSRS and suicidal ideation judged by the investigator to be serious/severe. TEAESIs involving terms of MDMA abuse, misuse, drug diversion, dependence or overdose were also collected.

### Reporting summary

Further information on research design is available in the Nature Portfolio Reporting Summary linked to this article.

## Online content

Any methods, additional references, Nature Portfolio reporting summaries, source data, extended data, supplementary information, acknowledgements, peer review information; details of author contributions and competing interests; and statements of data and code availability are available at 10.1038/s41591-023-02565-4.

## Supplementary information

Supplementary Information CONSORT checklist, MAPP2 study collaborators, Supplementary Methods, Supplementary Figs. 1 and 2, Supplementary Tables 1–9 and Supplementary References.

Reporting Summary

## Source data

Source Data Fig. 2 Statistical source data.

Source Data Fig. 3 Statistical source data.

## Data Availability

The data that support the findings of this study are available from the sponsor beginning 1 year after completion of the trial. However, restrictions apply to the availability of these data, which were used under license for the current study and so are not publicly available. Data are, however, available from the authors upon reasonable request and with the permission of the sponsor. All requests for raw and analyzed data are promptly reviewed to verify if the request is subject to any confidentiality obligations. Participant-related data not included in the paper were generated as part of clinical trials and may be subject to participant confidentiality. Any data that can be shared will be released via a data use agreement. Proposals should be directed to https:/maps.org/datause. Source data are provided with this paper.

## Code availability

Commercially available software (SAS version 9.4 or higher, SAS Institute) was used for analyses, in keeping with the statistical analysis plan.
