# Author Correction: MDMA-assisted therapy for moderate to severe PTSD: a randomized, placebo-controlled phase 3 trial

**DOI:** 10.1038/s41591-024-03331-w

**Published:** 2024-10-07

**Authors:** Jennifer M. Mitchell, Marcela Ot’alora G, Bessel van der Kolk, Scott Shannon, Michael Bogenschutz, Yevgeniy Gelfand, Casey Paleos, Christopher R. Nicholas, Sylvestre Quevedo, Brooke Balliett, Scott Hamilton, Michael Mithoefer, Sarah Kleiman, Kelly Parker-Guilbert, Keren Tzarfaty, Charlotte Harrison, Alberdina de Boer, Rick Doblin, Berra Yazar-Klosinski, MAPP2 Study Collaborator Group, Charlotte Harrison, Berra Yazar-Klosinski

**Affiliations:** 1 Neuroscape, Department of Neurology, University of California, San Francisco, San Francisco, CA USA; 2 Department of Psychiatry and Behavioral Sciences, University of California, San Francisco, San Francisco, CA USA; 3 Department of Veterans Affairs, Research Service, San Francisco VA Medical Center, San Francisco, CA USA; 4 Aguazul-Bluewater, Inc., Boulder, CO USA; 5 Boston University School of Medicine, Boston, MA USA; 6 Wholeness Center, Fort Collins, CO USA; 7 Department of Psychiatry, New York University Grossman School of Medicine, New York, NY USA; 8 Zen Therapeutic Solutions, Mt. Pleasant, SC USA; 9 Nautilus Sanctuary, New York, NY USA; 10 Department of Family Medicine and Community Health, University of Wisconsin School of Medicine and Public Health, Madison, WI USA; 11 San Francisco Insight and Integration Center, San Francisco, CA USA; 12 New School Research, LLC, Los Angeles, CA USA; 13 MAPS Public Benefit Corporation, San Jose, CA USA; 14 Medical University of South Carolina, Charleston, SC USA; 15 Kleiman Consulting and Psychological Services, PC, Ivyland, PA USA; 16 KPG Psychological Services, LLC, Brunswick, ME USA; 17 University of Haifa, Haifa, Israel; 18 MAPS Israel, Hod Hasharon, Israel; 19 Tulip Medical Consulting, LLC, Port Townsend, WA USA; 20 Multidisciplinary Association for Psychedelic Studies (MAPS), San Jose, CA USA

**Keywords:** Drug development, Trauma

Correction to: *Nature Medicine* 10.1038/s41591-023-02565-4, published online 14 September 2023.

In the version of the article initially published, some members of the MAPP2 Study Collaborator Group were listed only in the Supplementary Information. Charlotte Harrison, Berra Yazar-Klosinski, Wael Garas, Darrick May, Cole Marta, Susan Walker, Elizabeth Nielson, Gregory Wells, Randall Brown, Revital Amiaz, Yair Wallach, Ray Worthy, Alia Lilienstein and Amy Emerson have now been added to the main list of collaborators in the HTML and PDF versions of the article.

