# Supplementary material for: MDMA-assisted therapy for moderate to severe PTSD: a randomized, placebo-controlled phase 3 trial
Source: Nat Med. 2023 Sep 14;29(10):2473–80. doi: 10.1038/s41591-023-02565-4 (PMC10579091; doi:10.1038/s41591-023-02565-4)
Supplement: Supplementary file 1 — CONSORT checklist, MAPP2 study collaborators, Supplementary Methods, Supplementary Figs. 1 and 2, Supplementary Tables 1–9 and Supplementary References. [file 41591_2023_2565_MOESM1_ESM.pdf]

nature medicine

Article

https://doi.org/10.1038/s41591-023-02565-4

MDMA-assisted therapy for moderate to severe PTSD: a randomized, placebo-controlled phase 3 trial

In the format provided by the authors and unedited

SUPPLEMENTARY APPENDIX

**Table of Contents**	**Page**
**CONSORT Checklist**	**2**
**MAPP2 Study Collaborators**	**4**
**Supplementary Methods**	**5**
**Supplementary Figures and Tables**	
**Figure S1.** Trial Procedure Timeline	**9**
**Figure S2.** Number of Participants Reporting Suicidal Ideation, as Measured with C-SSRS Throughout the Trial	**10**
**Table S1.** Trial Representativeness of PTSD Population	**11**
**Table S2.** Study Treatment Deviations	**12**
**Table S3.** Missing Data from mITT Set	**13**
**Table S4.** SDS Total Scores by Domains	**14**
**Table S5.** Covariate Effects on Primary Results	**15**
**Table S6.** Blinding Survey	**16**
**Table S7.** Participants with Cardiovascular TEAEs and TEAESIs	**17**
**Table S8.** Changes in Vital Signs During Experimental Sessions	**18**
**Table S9.** Participants with Psychiatric TEAEs and TEAESIs	**20**
**References**	**21**

CONSORT 2010 checklist of information to include when reporting a randomized trial

Section/Topic	Item No	Checklist item	Reported on page No
**Title and abstract**			
	1a	Identification as a randomized trial in the title	1; referred to as Phase 3 Trial
	1b	Structured summary of trial design, methods, results, and conclusions (for specific guidance see CONSORT for abstracts)	3
**Introduction**			
Background and objectives	2a	Scientific background and explanation of rationale	4-5
	2b	Specific objectives or hypotheses	38 (Online Methods)
**Methods**			
Trial design	3a	Description of trial design (such as parallel, factorial) including allocation ratio	35 (Online Methods)
	3b	Important changes to methods after trial commencement (such as eligibility criteria), with reasons	n/a
Participants	4a	Eligibility criteria for participants	35 (Online Methods); Supplementary, 6
	4b	Settings and locations where the data were collected	35 (Online Methods)
Interventions	5	The interventions for each group with sufficient details to allow replication, including how and when they were actually administered	37-38 (Online Methods); Supplementary, 6-7
Outcomes	6a	Completely defined pre-specified primary and secondary outcome measures, including how and when they were assessed	38 (Online Methods); Supplementary, 6-7
	6b	Any changes to trial outcomes after the trial commenced, with reasons	n/a
Sample size	7a	How sample size was determined	40 (Online Methods)
	7b	When applicable, explanation of any interim analyses and stopping guidelines	n/a
Randomization:			
Sequence generation	8a	Method used to generate the random allocation sequence	35-37 (Online Methods)
	8b	Type of randomization; details of any restriction (such as blocking and block size)	35-37 (Online Methods)
Allocation concealment mechanism	9	Mechanism used to implement the random allocation sequence (such as sequentially numbered containers), describing any steps taken to conceal the sequence until interventions were assigned	35-37 (Online Methods)
Implementation	10	Who generated the random allocation sequence, who enrolled participants, and who assigned participants to interventions	35-37 (Online Methods)
Blinding	11a	If done, who was blinded after assignment to interventions (for example, participants, care providers, those assessing outcomes) and how	35-37 (Online Methods)
	11b	If relevant, description of the similarity of interventions	37-38 (Online Methods)
Statistical methods	12a	Statistical methods used to compare groups for primary and secondary outcomes	40-41 (Online Methods)
	12b	Methods for additional analyses, such as subgroup analyses and adjusted analyses	41 (Online Methods)
**Results**			
Participant flow (a diagram is strongly recommended)	13a	For each group, the numbers of participants who were randomly assigned, received intended treatment, and were analyzed for the primary outcome	Figure 1

	13b	For each group, losses and exclusions after randomization, together with reasons	Figure 1; Supplementary, 13 (Table S3)
Recruitment	14a	Dates defining the periods of recruitment and follow-up	5
	14b	Why the trial ended or was stopped	n/a
Baseline data	15	A table showing baseline demographic and clinical characteristics for each group	Table 1
Numbers analyzed	16	For each group, number of participants (denominator) included in each analysis and whether the analysis was by original assigned groups	Figure 1; Supplementary, 13 (Table S3)
Outcomes and estimation	17a	For each primary and secondary outcome, results for each group, and the estimated effect size and its precision (such as 95% confidence interval)	6
	17b	For binary outcomes, presentation of both absolute and relative effect sizes is recommended	n/a
Ancillary analyses	18	Results of any other analyses performed, including subgroup analyses and adjusted analyses, distinguishing pre-specified from exploratory	7-8
Harms	19	All important harms or unintended effects in each group (for specific guidance see CONSORT for harms)	8-10; Table 2; Supplementary: Figure S2; Tables S7-S9
**Discussion**			
Limitations	20	Trial limitations, addressing sources of potential bias, imprecision, and, if relevant, multiplicity of analyses	13-14
Generalizability	21	Generalizability (external validity, applicability) of the trial findings	10-11
Interpretation	22	Interpretation consistent with results, balancing benefits and harms, and considering other relevant evidence	11-12
**Other information**			
Registration	23	Registration number and name of trial registry	3 (Abstract)
Protocol	24	Where the full trial protocol can be accessed, if available	18
Funding	25	Sources of funding and other support (such as supply of drugs), role of funders	41-42 (Online Methods)

MAPP2 Study Collaborators

We acknowledge the oversight of the principal investigators and study therapists for their expert treatment of participants. We also thank the study coordinators, medical providers, night attendants, Data Monitoring Committee members, Independent and Adherence Raters, and MAPS PBC staff for their efforts. We would like to acknowledge the following leaders from the MAPP2 Study Collaborators Group:

Collaborator	Site/Affiliation	Contribution
Charlotte Harrison, BA	MAPS Public Benefit Corporation, San Jose, CA, USA	Sponsor Clinical Operations Director
Berra Yazar-Klosinski, PhD	MAPS Public Benefit Corporation, San Jose, CA, USA	Sponsor Chief Scientific Officer
Wael Garas, MD	Aguazul-Bluewater Inc., Boulder, CO, USA	Co-Investigator
Darrick May, MD	Wholeness Center, Fort Collins, CO, USA	Co-Investigator
Cole Marta, MD	New School Research LLC, Hollywood, CA, USA	Co-Investigator
Susan Walker, MD	Trauma Research Foundation, Boston, MA, USA	Co-Investigator
Elizabeth Nielson, PhD	Nautilus Sanctuary, New York, NY, USA; Columbia University Department of Psychiatry and New York State Psychiatric Institute, New York, NY, USA	Co-Investigator
Gregory Wells, PhD	San Francisco Insight and Integration Center, San Francisco, CA, USA	Co-Investigator
Randall Brown, MD, PhD	Department of Family Medicine and Community Health, University of Wisconsin School of Medicine and Public Health, Madison, WI, USA	Co-Investigator
Revital Amiaz, MD	Chaim Sheba Medical Center, Tel Ha'Shomer, Israel	Investigator
Yair Wallach, MD	Merhavim - The Medical Center for the Treatment of Mind and Soul in Beer Yaakov, Be'er Ya'akov, Israel	Investigator
Ray Worthy, MD	Ray Worthy Psychiatry LLC, New Orleans, LA, USA	Investigator
Alia Lilienstein, MD, MPH	MAPS Public Benefit Corporation, San Jose, CA, USA,	Sponsor Medical Director
Amy Emerson	MAPS Public Benefit Corporation, San Jose, CA, USA	Sponsor Designee and representative of the MAPP2 Study Collaborators Group (email: amy@mapsbcorp.com)

SUPPLEMENTARY METHODS

Review boards/institutions

The MAPP2 study protocol was approved by the following institutional review boards: Western Copernicus Group Independent Review Board (Puyallup, WA, USA), Helsinki Committee of Merhavim Medical Center for Brain and Mental Health Treatment (Beer Yaakov, Israel), and Helsinki Committee of The Chaim Sheba Medical Center (Ramat Gan, Israel).

The following institutional review board sites deferred to the Western Copernicus Group Independent Review Board for approving the MAPP2 study protocol: University of California, San Francisco Human Research Protection Program Institutional Review Board (San Francisco, CA, USA), University of Wisconsin Madison, Health Sciences Institutional Review Board (Madison, WI, USA), New York University School of Medicine Institutional Review Board (New York, NY, USA).

Inclusion criteria

Individuals were eligible to enroll in the study if they were ≥18 years old, fluent in the language used by the study site, able to swallow pills, had a contactable support person, used adequate birth control (if applicable), agreed to follow lifestyle modifications, and agreed to the recording of all study visits.

Additionally, individuals were eligible for the study if they met DSM-5 criteria for current post-traumatic stress disorder (PTSD) for ≥6 months, had experienced at least moderate PTSD symptoms in the month before baseline, and had a Clinician-Administered PTSD Scale for DSM-5 (CAPS-5) total severity score of ≥28. Individuals with medical conditions such as hypertension, asymptomatic hepatitis C virus, diabetes mellitus, hyperthyroidism, and glaucoma were eligible, providing the condition was well managed and mild. Individuals with current mild alcohol or cannabis use disorders, or with a moderate alcohol or cannabis-use disorder in early remission for ≥3 months prior to enrollment, were also eligible.

Exclusion criteria

Individuals were ineligible to enroll if they were unable to give informed consent. Individuals were also excluded for a history of or current primary psychotic disorder, bipolar I disorder, dissociative identity disorder, eating disorder with active purging, major depressive disorder with psychotic features, personality disorders, severe alcohol or cannabis use disorder (also moderate if not in remission), any substance use disorder other than cannabis or alcohol within 12 months prior to enrollment, use of ecstasy (material represented as containing MDMA) more than 10 times within the last 10 years or at least once within 6 months of the first experimental session, serious imminent suicide risk, and any medical condition that could make receiving a sympathomimetic drug harmful due to increased blood pressure and heart rate, including uncontrolled hypertension, history of arrhythmia, or marked baseline prolongation of QT and/or QTc interval. Individuals were also excluded if they weighed ≤48 kg, were pregnant or nursing, were engaged in electroconvulsive therapy or ketamine-assisted therapy, or had used ketamine within 12 weeks of enrollment.

Study drug

The study drug (3,4-methylenedioxymethamphetamine [MDMA]) was manufactured in accordance with Current Good Manufacturing Practice (cGMP) standards by Sharp Clinical Services, LLC (Bethlehem, PA, USA) and Recipharm (Queenborough, UK). MDMA was encapsulated with excipients in hydroxypropyl methylcellulose.

Trial procedures

Following screening procedures, participants attended a total of three preparation sessions, three experimental sessions, nine integration sessions, and four endpoint assessments over the course of 18 weeks, concluding with a final study termination visit **(Figure S1)**. Full details for trial procedures are below.

Preparation sessions (visits 1, 2, and 4) focused on establishing a therapeutic alliance and providing guidance on how the participant could remain present with the memories and emotions that could arise during treatment. All eligible participants were required to undergo medically supervised tapering and discontinuation of psychiatric medications for a minimum of five half-lives plus 1 week before baseline assessments. After washout, participants completed baseline CAPS-5 and Sheehan Disability Scale (SDS) assessments (T1), conducted by an independent

rater, at visit 3 (**Figure S1**). At the end of the preparation period (visit 4), final eligibility was assessed using the Mini-International Neuropsychiatric Interview (MINI), the Structured Clinical Interview for DSM-5 Personality Disorders (SCID-5-PD), the Dissociative Disorders Interview Schedule (DDIS), and the Columbia Suicide Severity Rating Scale (C-SSRS), and enrollment was confirmed prior to randomization.

The treatment period (~12 weeks) consisted of three 8-hour experimental sessions with either MDMA or inactive placebo (visits 5, 10, 15) spaced 3–5 weeks apart, both in conjunction with therapy. Following a 10-hour fast, experimental sessions began with a C-SSRS assessment and vital sign measurements (baseline blood pressure, body temperature, and pulse) immediately before the initial drug dose. Participants received a split dose of MDMA (80 mg initial dose in experimental session 1, and 80 or 120 mg initial dose in subsequent sessions) or placebo, followed by an additional half-dose of MDMA (40 mg in experimental session 1, and 40 or 60 mg in subsequent sessions) or placebo approximately 1.7 hours later, unless tolerability issues emerged or the participant declined. The study drug was taken orally with water or electrolyte-containing fluid. Vital signs were measured during the experimental sessions. Most participants stayed overnight (n=74: MDMA-AT, n=39; placebo with therapy, n=35) at the study site with night attendants, but a subset (n=30: MDMA-AT, n=14; placebo with therapy, n=16) went home with a support person.

Each experimental session was followed by three 90-minute integration sessions (visits 6, 7, 9, 11, 12, 14, 16, 17, and 18) of non-drug therapy to support the participant in processing and understanding their experience. The first integration session took place on the morning after the experimental session and was followed by phone check-ins every other day for 2 weeks. The second and third integration sessions occurred over 3–4 weeks following the first integration session. Suicidal ideation/behavior (as measured by the C-SSRS), adverse events (AEs), and concomitant medications were tracked throughout the study.

Independent raters conducted CAPS-5 and SDS outcome assessments at baseline (visit 3), after the first two experimental sessions (visits 8 and 13), and 6–8 weeks after the third experimental session (visit 19; 18 weeks post-baseline) via live video interviews.

Therapy Teams and Training

Study sites were required to include: one person licensed to manage and administer controlled substances; a physician to assess participant safety at Screening; one or more two-person therapy pairs, male/female preferred; one person per therapy pair licensed to provide psychotherapy according to state and local requirements (if one person on the therapy pair was unlicensed, they worked under the direction of the licensed pair member).

All therapy teams were trained in a multi-week program prior to the study. Therapy team members received specific training in the MDMA-AT method, protocol, and latest version of the Investigator's Brochure. Therapy training included reading the Treatment Manual, completing an online training module, and participating in an in-person training program that included watching and discussing videos of Experimental Sessions. The final part of training included close supervision from the training team throughout the enrollment of a therapy team member's first study participant, with additional supervision for subsequent enrollments provided, if deemed necessary by the training team or Adherence Raters, or at the request of the therapy team member.

Exploratory outcomes

Exploratory outcome measurements included characterization of the treatment response and differences between the treatment groups by demographics and characteristics.

Responder analyses were based on categorical diagnostic assessment data and the CAPS-5 total severity score assessment. PTSD severity was defined using the CAPS-5 total severity score as follows: asymptomatic (0–10), mild (11–22), moderate (23–34), severe (35–46), extreme (47+). A ≥10-point reduction in CAPS-5 total severity score was considered to be clinically meaningful as agreed upon with the US Food and Drug Administration (FDA) through a Special Protocol Assessment. Four responder categories were derived and compared at each post-experimental session visit using CAPS-5 scores. These categories were: non-responder (<10-point reduction from baseline), responder (≥10-point reduction from baseline), loss of diagnosis (≥10-point reduction from baseline and no longer meeting PTSD diagnostic criteria), and remission (CAPS-5 Total Severity Score of 11 or less and no longer meeting PTSD diagnostic criteria).

In additional exploratory analyses, 13 covariates were assessed in the model, with alpha set at 0.0499: age, sex (self-reported), prior use of selective serotonin reuptake inhibitors (SSRIs), work disability, disease severity, PTSD duration, dissociative subtype, overnight site stay, site ID, moderate depression (as measured by the Beck Depression Inventory [BDI-II]), severe adverse childhood experiences, and moderate alcohol and substance use disorder risk (as measured by the Drug Use Disorders Identification Test and Alcohol Use Disorders Identification Test).

Safety Objectives

Columbia Suicide Severity Rating Scale

The C-SSRS was clinician-administered at each study visit to assess suicidal ideation, ideation intensity, and suicidal behavior. Two versions were used in this study: the Lifetime C-SSRS was conducted for study eligibility, and the Since Last Visit C-SSRS was conducted at all visits post-screening.[1,2] The C-SSRS Scoring and Data Analysis Guide was followed for C-SSRS analysis. Positive suicidal ideation was identified when a participant answered “yes” to any one of the five suicidal ideation questions (categories 1–5) on the C-SSRS (i.e., a score of >0 for suicidal ideation). Serious suicidal ideation was defined as a suicidal ideation score of 4 or 5. Treatment-emergent suicidal ideation and behavior was defined as any increase in C-SSRS score after the first dose of the study drug. If a participant had positive suicidal ideation, extra visits were conducted to follow suicidal ideation to resolution during which C-SSRS was administered.

Statistical analysis

The statistical analysis plan was guided by the ICH E9 (R1) guidelines[3], which describe the use of estimands and sensitivity analyses to measure the effects of a drug if taken as directed (‘de jure,’ assessment of efficacy using an initially randomized treatment estimand) and if taken as assigned, regardless of adherence (‘de facto,’ assessment of effectiveness using a treatment policy estimand). The de jure dataset did not include outcome measurements taken after treatment discontinuation in the analysis of treatment efficacy, and these were considered missing at random. The de facto estimand, a supportive sensitivity analysis, included CAPS-5 data collected after treatment discontinuations. One participant discontinued after experimental session 1 and declined outcome data collection; this participant was excluded from primary and secondary efficacy analyses per the modified Intent to Treat analysis set, which required exposure to MDMA or placebo and at least one follow-up CAPS-5 assessment (**Figure 1 and Table S3**). To test the impact of the missing at random assumption, tipping point and worst-case sensitivity analyses were conducted. Statistics for the primary and key secondary efficacy comparisons (CAPS and SDS, respectively) are reported as least-squares mean change with 95% confidence intervals and *P* values from the results of the mixed model repeated measures (MMRM) analysis. Missing data was not imputed in the primary analysis. Between-group effect size and MDMA-AT within-group effect size were calculated with Cohen’s d.

A tipping point sensitivity analysis was conducted to estimate the treatment difference in the missing CAPS-5 scores where model results became statistically non-significant. This sensitivity analysis assumes that all missing CAPS-5 data are Missing Not at Random (MNAR). Multiple imputation was used to impute missing CAPS-5 Visit 13 and/or Visit 19 data. The monotone regression procedure was used in SAS PROC MI to impute missing Visit 13 CAPS-5, using Visit 8 CAPS-5, dissociative subtype, site, and treatment as covariates. Missing Visit 19 CAPS-5 were then imputed in a similar manner, replacing Visit 8 CAP-5 with Visit 13 CAPS-5 as a predictor. The imputed MDMA-AT results were then penalized by Δ and estimates were generated from these penalized datasets. Final results for Δ penalized datasets were combined from 50 imputation datasets by applying Rubin’s Rules. Delta were increased in increments of x until the final model result p-value crosses the threshold of 0.0499.

To begin the tipping point analysis, the minimum shift needed to make the result non-significant must be determined. This is defined as the shift parameter (Δ). For these analyses, the CAPS-5 shift parameter (Δ) was determined to be 36 for MDMA treated participants with missing data. This large CAPS-5 shift parameter indicates the degree of shift in the missing data that would be needed to significantly reduce the overall effect in MDMA treated participants. Since there is only 1 MDMA treated participant with missing Visit 19 CAPS-5, there was no tipping point from penalizing the MDMA-AT group alone. Thus, the shift parameter (Δ) for the participants with missing data in the placebo group was then changed from no shift to various levels of reduction (ie, the negative shift values). With each larger reduction in score the participants in the placebo group with missing data were being shifted in a more favorable direction for response. With these shifts, the results continued to be significant even with a shift of 27 for the placebo group, but when the shift value was then made to be – (28) the p value crossed the

threshold of 0.0499 (the significance level set for the primary endpoint). The goal of the tipping point analysis was to assess whether the missingness in the data could have had an effect on the primary outcome result. Based on the results of the tipping point analysis, it can be concluded that for the missing CAPS-5 measures to have had a meaningful effect on the primary analysis, the unobserved results would have had to be extreme and outside realistic expectations.

SUPPLEMENTARY FIGURES

Figure S1. Trial Procedure Timeline. Following screening procedures, participants attended a total of three preparation sessions, three experimental sessions, nine integration sessions, and four endpoint assessments (T1–T4) over the course of 18 weeks, concluding with a final study termination visit. During the 8-hour experimental sessions, participants received an initial dose of placebo or MDMA (80 mg at V5; 80 or 120 mg at V10 and V15) followed by an additional half-dose (40 mg at V5; 40 or 60 mg at V10 and V15) approximately 2 hours later. CAPS-5 and SDS outcome assessments (T1–T4) were conducted at V3, V8, V13, and V19. Refer to the **Supplementary Methods** for a full description of trial procedures. [a]Participants tapered off any prohibited psychiatric medications during the preparation period. Abbreviations: CAPS-5, Clinician-Administered PTSD Scale for DSM-5; MDMA, 3,4-methylenedioxymethamphetamine; MDMA-AT, MDMA-assisted therapy; SDS, Sheehan Disability Scale; T, timepoint of endpoint assessment; T1, baseline; T2, after first experimental session; T3, after second experimental session; T4, after third experimental session and 18 weeks post-baseline; V, visit. Illustrations included in this figure publicly available from GoodReports (goodreports.org).

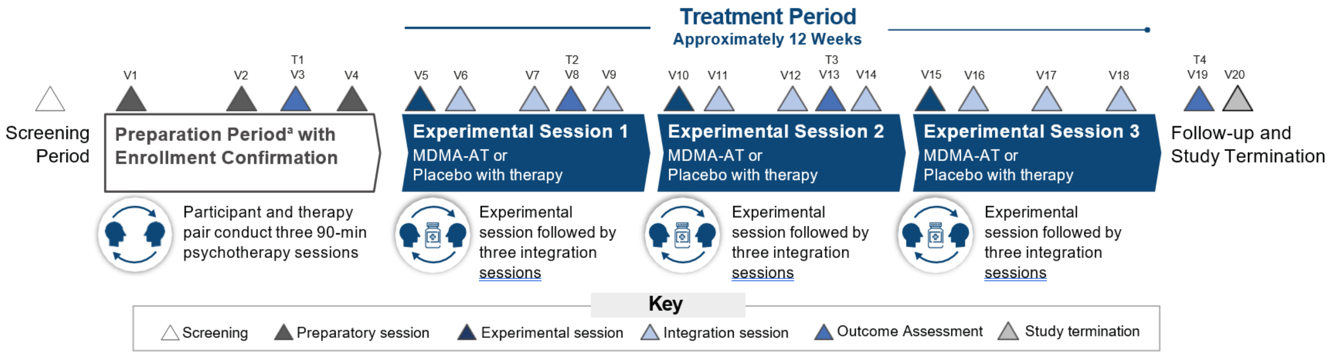

Figure S2. Number of Participants Reporting Suicidal Ideation, as Measured with the C-SSRS Throughout the Trial. C-SSRS scores range from 0 (no ideation) to 5; a score of 4 or 5 indicates active suicidal ideation with at least some intent to act. The number of participants with any positive ideation (score >0) is indicated by the colored bars and noted in the table below the graph. The number of events where the participant had a score of 4 or 5 is given within parentheses in the table. [a]If a participant had positive suicidal ideation, extra visits were conducted to follow suicidal ideation to resolution during which the C-SSRS was administered. Abbreviations: C-SSRS, Columbia Suicide Severity Rating Scale; MDMA-AT, 3,4-methylenedioxymethamphetamine-assisted therapy.

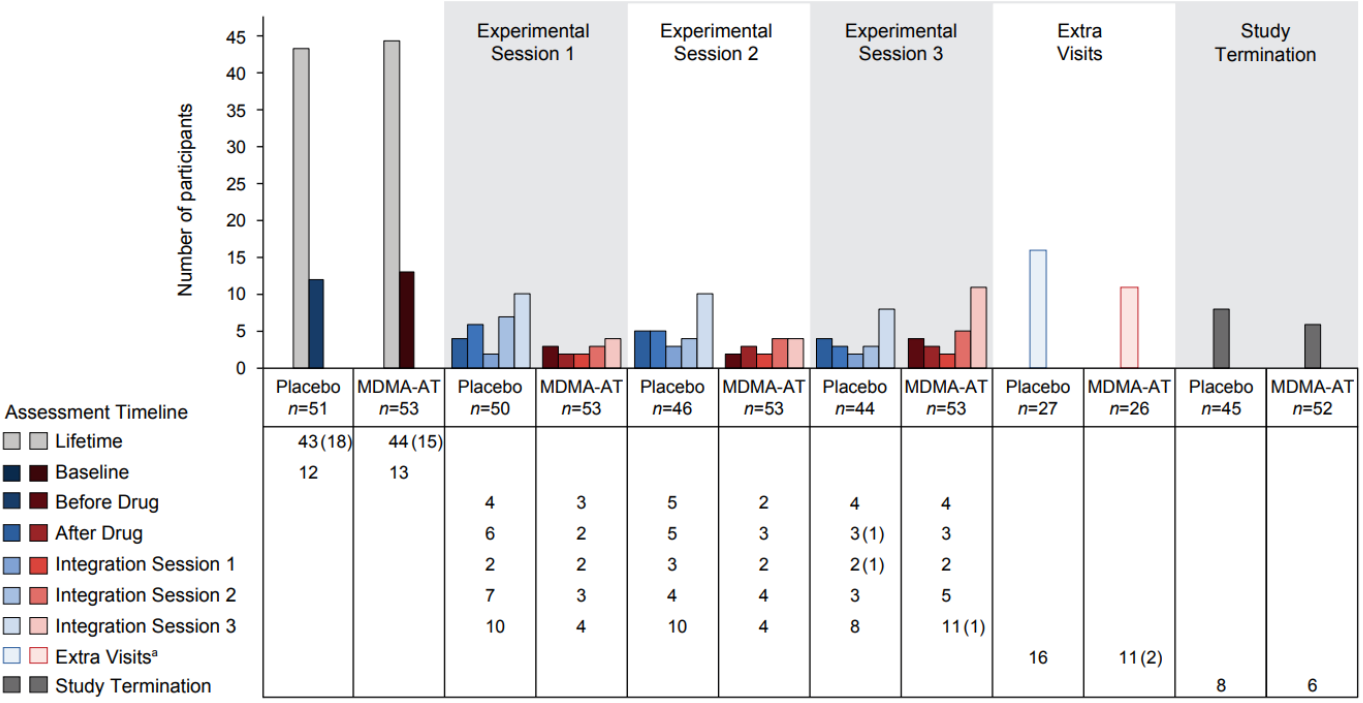

Assessment Timeline	Placebo *n*=51	MDMA-AT *n*=53	Placebo *n*=50	MDMA-AT *n*=53	Placebo *n*=46	MDMA-AT *n*=53	Placebo *n*=44	MDMA-AT *n*=53	Placebo *n*=27	MDMA-AT *n*=26	Placebo *n*=45	MDMA-AT *n*=52
Lifetime	43 (18)	44 (15)										
Baseline	12	13										
Before Drug			4	3	5	2	4	4				
After Drug			6	2	5	3	3 (1)	3				
Integration Session 1			2	2	3	2	2 (1)	2				
Integration Session 2			7	3	4	4	3	5				
Integration Session 3			10	4	10	4	8	11 (1)				
Extra Visits[a]									16	11 (2)		
Study Termination											8	6

SUPPLEMENTARY TABLES

Table S1. Trial Representativeness of PTSD Population

Category	Considerations
**Condition under investigation**	Post-traumatic stress disorder (PTSD)
**Sex and gender**	PTSD prevalence is twice as high among women (8%) versus men (4%).[4] Gender-diverse and transgender individuals are at a higher risk of developing PTSD.[5]
**Age**	PTSD may impact individuals throughout their course of life. Traumatic events that lead to PTSD may begin in early childhood; as many as 61% of US adults report having experienced developmental traumas, including exposure to abuse, neglect, and chronic poverty.[6] PTSD can be a chronic condition, with as many as 23% of individuals not meeting recovery criteria after 10 years.[7]
**Race or ethnic group**	Discrimination is a chronic and ongoing stressor that can contribute to the incidence of PTSD in marginalized groups, including specific minority races and ethnicities.[8] Black Americans have a higher prevalence of PTSD (8.7%) compared with White (7.4%), Hispanic (7.0%), and Asian (4.0%) Americans.[9]
**Geography**	PTSD poses a global mental health challenge, with approximately 3.9% of the worldwide population affected.[10] Estimates vary widely by country and region, with lifetime prevalence of 5% in high-income countries, 2.3% in middle-income countries, and 2.1% in low-income countries.[10] Prevalence estimates vary in part due to the heterogeneity of the populations assessed, differences in time periods evaluated, and under- or misdiagnosis.[11]
**Other considerations**	Nearly 80% of individuals with a diagnosis of PTSD meet criteria for at least one other comorbid condition, such as depression or substance-use disorder.[12] Those with PTSD may also experience multiple sources of trauma.[13] Additionally, sociopolitical issues contributing to material hardship,[14] homelessness,[15] and lack of social support structures[16] may further predispose trauma-exposed individuals to develop PTSD. Further, PTSD is higher among certain groups, such as veterans, when compared to civilians.[17]
**Overall representativeness of this trial**	The current trial enrolled a high proportion of women and is consistent with the 2:1 ratio of women to men seen in PTSD diagnostic prevalence. A high percentage of participants (27%) self-identified as Hispanic or Latino and 34% were non-White. In terms of lifetime burden, the mean duration of PTSD among the overall sample was 16.2 years, and 89% of participants had been exposed to some form of developmental trauma.

Table S2. Study Treatment Deviations. For three participants, the MDMA dose was not escalated in experimental sessions 2 and 3. Two participants experienced MDMA dose administration errors during the study.

**Dose escalations**			
**Treatment**	**Timepoint**	**Description**	
MDMA (n=3)	Visit 15 (experimental session 3)	Participant took an escalation dose at visit 10, but chose not to escalate the dose at visit 15.	
	Visits 10 and 15 (experimental sessions 2 and 3)	Participant experienced transient hypertension during visit 5 (experimental session 1). Therefore, the dose was not escalated in experimental sessions 2 and 3 for this participant per medical monitor and primary investigator's judgement.	
	Visits 10 and 15 (experimental sessions 2 and 3)	Participant received an initial dose of 120 mg at visits 10 and 15, but did not receive supplemental doses at these visits.	
**Dosing errors**			
**Treatment**	**Timepoint**	**Protocol deviation classification**	**Description**
MDMA (n=2)	Visit 5 (experimental session 1)	Major	Participant was incorrectly given an initial dose of 40 mg and a supplemental dose of 80 mg. Participant received a total of 120 mg during this session.
	Visit 15 (experimental session 3)	Minor	Participant was incorrectly given an initial dose of 100 mg and a supplemental dose of 40 mg. Participant received a total of 140 mg during this session.
Abbreviation: MDMA, 3,4-methylenedioxymethamphetamine.			

Table S3. Missing Data from mITT Set. Missing data from the mITT set based on reasons for post-randomization withdrawal.

Reason for study termination	Treatment (sample size)	Summary of missing data and inclusion in de jure estimand		
		**Reason**	**Missed visit(s)**	**Included in de jure estimand**
Dropout	MDMA-AT (n=1)	Participant chose to discontinue treatment	T4	T1, T2, T3
	Placebo with therapy (n=4)	Participant chose to discontinue treatment	T2, T3, T4	Not in mITT
		Participant chose to discontinue treatment; participant declined to participate	T3, T4	T1, T2
		Participant chose to discontinue treatment; adverse event – passive suicidal ideation; withdrawal of consent	T3, T4	T1, T2
		Participant chose to discontinue treatment; lost to follow-up	T4	T1, T2, T3
Post-randomization early termination	MDMA-AT	None observed	–	–
	Placebo with therapy (n=4)	Participant chose to discontinue treatment; participant declined to participate	T3, T4	T1, T2
		Participant chose to discontinue treatment	T3, T4	T1, T2
		Adverse event – abdominal pain	T3	T1, T2
		Participant chose to discontinue treatment as they believed they were receiving placebo	T3	T1, T2

Abbreviations: MDMA-AT, 3,4-methylenedioxymethamphetamine-assisted therapy; mITT, modified intent to treat; T, timepoint of endpoint assessment; T1, baseline; T2, after first experimental session; T3, after second experimental session; T4, after third experimental session and 18 weeks post-baseline.

Table S4. SDS Total Scores by Domains. SDS total scores by domains at baseline (MDMA-AT, n=53; placebo with therapy, n=50) and 18 weeks post-baseline (MDMA-AT, n=52; placebo with therapy, n=42).

	**Family life/home**		**Social/leisure activities**		**Work/school**	
	**MDMA-AT**	**Placebo with Therapy**	**MDMA-AT**	**Placebo with Therapy**	**MDMA-AT**	**Placebo with Therapy**
Baseline score, mean (SD)	5.1 (2.7)	5.6 (2.0)	6.2 (2.3)	6.5 (2.0)	6.8 (2.6)	6.3 (2.5)
18 weeks post-baseline, mean (SD)	2.2 (2.9)	4.1 (2.9)	2.6 (3.0)	3.9 (3.1)	3.4 (3.5)	3.9 (3.3)

Abbreviations: MDMA-AT, 3,4-methylenedioxymethamphetamine-assisted therapy; SD, standard deviation; SDS, Sheehan Disability Scale.

Table S5. Covariate Effects on Primary Results. Additional baseline covariates were assessed in an MMRM model for effects on the primary efficacy outcome.

**MMRM results for fixed effects**		
**Effect**	**F-value**	**Pr > F**
Treatment	12.42	0.0007
Baseline CAPS-5 score	2.53	0.1154
Study visit	51.46	<0.0001
Treatment × study visit	3.32	0.0407
Dissociative subtype	3.39	0.0691
Study site	0.93	0.5252
**Covariate effects on primary results (exploratory)**		
**Variable**	***P*-value main effect**	***P*-value interaction with treatment**
Age	0.3184	0.9878
Sex assigned at birth (self-report)	0.0109	0.6136
Disabled from work	0.4700	0.9821
Disease severity	0.7777	0.7473
SSRI history	0.8217	0.0177
PTSD duration	0.8323	0.8409
CAPS-5 dissociative subtype	0.0709	0.9668
BDI-II (≥23 and <23)	0.0229	0.3298
ACE (≥4 and <4)	0.5239	0.2065
AUDIT (≥5 and <5)	0.9235	0.1028
DUDIT (≥5 and <5)	0.4492	0.1969
Site ID	0.4384	0.2012
Overnight/no overnight stay	0.7275	0.6077
Abbreviations: ACE, adverse childhood experience; AUDIT, Alcohol Use Disorders Identification Test; BDI, Beck Depression Inventory; CAPS-5, Clinician-Administered PTSD Scale for DSM-5; DUDIT, Drug Use Disorders Identification Test; MMRM, mixed model repeated measures; Pr > F, p-value of the F statistic; no correction for multiple comparisons. PTSD, post-traumatic stress disorder; SSRI, selective serotonin reuptake inhibitor.		

Table S6. Blinding Survey. A blinding survey was conducted at study termination (visit 20) for participants.

Belief on study drug received, n (%)	MDMA-AT (n=52)	Placebo with Therapy (n=44)
MDMA – I am positive	41 (78.8)	2 (4.5)
MDMA – I think	8 (15.4)	7 (15.9)
Placebo – I am positive	1 (1.9)	19 (43.2)
Placebo – I think	0	14 (31.8)
Cannot tell	2 (3.8)	2 (4.5)
Abbreviations: MDMA, 3,4-methylenedioxymethamphetamine; MDMA-AT, MDMA-assisted therapy		

Table S7. Participants with Cardiovascular TEAEs and TEAESIs

	MDMA-AT (n=53)	Placebo with Therapy (n=51)
**Cardiac TEAEs**		
Overall	7 (13.2)	1 (2.0)
Palpitations	5 (9.4)	1 (2.0)
Tachycardia	2 (3.8)	0
Postural orthostatic tachycardia syndrome	1 (1.9)	0
**Vascular TEAEs**		
Overall	7 (13.2)	2 (3.9)
Flushing	3 (5.7)	1 (2.0)
Peripheral coldness	2 (3.8)	2 (3.9)
Hot flush	1 (1.9)	0
Hypertension	1 (1.9)[a]	0
**Cardiac TEAESIs[b]**		
Palpitations	4[c]	1

[a]Characterized as moderate hypertension; the participant experienced systolic/diastolic (mmHg) blood pressure readings as follows: 138/88 (Predose), 176/106 (Interim), 170/100 (additional measurement), 140/88 (Endpoint). [b]Per protocol, TEAESIs included any events involving cardiac function that could be indicative of QT interval prolongation or cardiac arrhythmias, including torsade de pointes, sudden death, ventricular extrasystoles, ventricular tachycardia, ventricular fibrillation and flutter, non-postural syncope, and seizures. Data are presented on a participant level for events occurring after treatment initiation. [c]One participant that had a TEAE for palpitations did not meet criteria for a TEAESI based on further assessment via electrocardiogram.
Abbreviations: MDMA-AT, 3,4-methylenedioxymethamphetamine-assisted therapy; TEAE, treatment-emergent adverse event; TEAESI, treatment-emergent adverse event of special interest.

Table S8. Changes in Vital Signs During Experimental Sessions. Vital sign measurements were collected during the experimental sessions at predose (~5 min prior to initial dose), interim (~2 h post-initial dose), and endpoint (~8 h post-initial dose). Doses of MDMA were as follows: experimental session 1 (80 mg + 40 mg); experimental session 2 (120 mg + 60 mg); and experimental session 3 (120 mg + 60 mg). Transient increases in vital signs were observed in the MDMA-AT group, as expected based on prior data from phase 2 and 3 studies.

	**MDMA-AT (n=53)**	**Placebo with Therapy (n=51)**
**Mean Values: Experimental session 1 (visit 5)**		
Predose, n	53	51
Systolic BP (mmHg), mean (SD)	124.4 (14.86)	121.1 (13.74)
Diastolic BP (mmHg), mean (SD)	80.4 (10.28)	79.6 (10.07)
Pulse (bpm), mean (SD)	72.3 (12.06)	73.9 (13.30)
Body temperature (°C), mean (SD)	36.4 (0.68)	36.5 (0.49)
Interim, n	53	51
Systolic BP (mmHg), mean (SD)	137.2 (15.06)	118.7 (12.78)
Diastolic BP (mmHg), mean (SD)	86.3 (9.71)	76.2 (9.12)
Pulse (bpm), mean (SD)	84.5 (17.62)	69.4 (9.87)
Body temperature (°C), mean (SD)	36.7 (0.56)	36.5 (0.61)
Endpoint, n	53	50
Systolic BP (mmHg), mean (SD)	124.3 (14.01)	118.5 (13.62)
Diastolic BP (mmHg), mean (SD)	79.6 (9.13)	78.3 (9.08)
Pulse (bpm), mean (SD)	82.2 (14.96)	72.3 (9.77)
Body temperature (°C), mean (SD)	36.7 (0.70)	36.7 (0.57)
**Mean Values: Experimental session 2 (visit 10)**		
Predose, n	53	46
Systolic BP (mmHg), mean (SD)	125.8 (11.42)	119.9 (14.19)
Diastolic BP (mmHg), mean (SD)	82.5 (8.74)	77.8 (11.16)
Pulse (bpm), mean (SD)	73.7 (10.90)	73.3 (13.94)
Body temperature (°C), mean (SD)	36.5 (0.53)	36.4 (0.58)
Interim, n	52	46
Systolic BP (mmHg), mean (SD)	143.0 (18.34)	121.6 (12.47)
Diastolic BP (mmHg), mean (SD)	88.7 (10.10)	81.0 (10.50)
Pulse (bpm), mean (SD)	91.7 (17.00)	70.9 (11.77)
Body temperature (°C), mean (SD)	36.7 (0.58)	36.5 (0.56)
Endpoint, n	52	46
Systolic BP (mmHg), mean (SD)	123.1 (12.62)	116.9 (12.45)
Diastolic BP (mmHg), mean (SD)	80.5 (8.51)	77.7 (8.66)
Pulse (bpm), mean (SD)	84.3 (14.81)	72.2 (11.24)
Body temperature (°C), mean (SD)	36.7 (0.54)	36.5 (0.61)
**Mean Values: Experimental session 3 (visit 15)**		
Predose, n	53	43
Systolic BP (mmHg), mean (SD)	126.2 (13.32)	119.2 (14.35)
Diastolic BP (mmHg), mean (SD)	81.5 (9.92)	78.1 (9.26)
Pulse (bpm), mean (SD)	73.6 (12.55)	70.7 (12.20)

Body temperature (°C), mean (SD)	36.5 (0.62)	36.5 (0.50)
Interim, n	53	43
Systolic BP (mmHg), mean (SD)	143.2 (15.29)	118.8 (12.96)
Diastolic BP (mmHg), mean (SD)	89.0 (10.62)	77.1 (10.43)
Pulse (bpm), mean (SD)	94.6 (20.05)	67.5 (11.53)
Body temperature (°C), mean (SD)	36.8 (0.52)	36.6 (0.45)
Endpoint, n	53	42
Systolic BP (mmHg), mean (SD)	124.6 (13.90)	117.9 (13.18)
Diastolic BP (mmHg), mean (SD)	80.9 (8.60)	77.6 (10.63)
Pulse (bpm), mean (SD)	86.0 (14.44)	71.4 (11.69)
Body temperature (°C), mean (SD)	36.7 (0.68)	36.6 (0.43)
**Post-Dose Extremes During Experimental Sessions 1-3**		
Systolic BP ≥180 mmHg, number of events	1	0
Diastolic BP ≥110 mmHg, number of events	4	0
Pulse >110 bpm, number of events	26	0
Body temperature ≥38 °C, number of events	4	0
Abbreviations: BP, blood pressure; bpm, beats per minute; MDMA-AT, 3,4-methylenedioxymethamphetamine-assisted therapy; SD, standard deviation.		

Table S9. Participants with Psychiatric TEAEs and TEAESIs.

	MDMA-AT (n=53)	Placebo with Therapy (n=51)
**Psychiatric TEAEs[a], n (%)**		
Overall psychiatric TEAEs	44 (83.0)	37 (72.5)
Suicidal ideation	18 (34.0)	21 (41.2)
Insomnia	19 (35.8)	15 (29.4)
Anxiety	15 (28.3)	12 (23.5)[b]
Depressed mood	5 (9.4)	6 (11.8)
Restlessness	8 (15.1)	2 (3.9)
Bruxism	7 (13.2)	1 (2.0)
Nightmares	4 (7.5)	3 (5.9)
Emotional disorder	3 (5.7)	2 (3.9)
Irritability	3 (5.7)	2 (3.9)[b]
Panic attack	3 (5.7)	1 (2.0)
Binge eating	0	3 (5.9)
Dissociation	3 (5.7)	0
**Severe psychiatric TEAEs, n (%)**		
Overall severe psychiatric TEAEs	3 (5.7)	2 (3.9)
Agitation	0	1 (2.0)
Anxiety	0	1 (2.0)
Dissociation	1 (1.9)	0
Flashback	1 (1.9)	0
Grief reaction	1 (1.9)	0
**Suicidality TEAESIs[c], n**		
Suicidal ideation[d]	2	1
Non-suicidal self-injurious behavior	1	1
Trichotillomania	0	1

[a]Psychiatric TEAEs that occurred in >5% of participants in either group. [b]One participant in the placebo with therapy group reported adverse events of mild anxiety and irritability which were retrospectively identified after data lock (not captured in the current table). [c]Per protocol, TEAESIs for suicide risk were defined as suicides, suicide attempts, self-harm associated with suicidal ideation, suicide ideation assessed as a 4 or 5 on the C-SSRS, and suicide ideation judged to be serious/severe by the investigator. Data are presented on a participant level for events occurring after treatment initiation. [d]Two participants experiencing suicidal ideation also experienced non-suicidal self-injurious behavior (MDMA-AT, n=1) or trichotillomania (placebo, n=1).
Abbreviations: C-SSRS, Columbia Suicide Severity Rating Scale; MDMA-AT, 3,4-methylenedioxymethamphetamine-assisted therapy; TEAE, treatment-emergent adverse event; TEAESI, treatment-emergent adverse event of special interest.

References

1. Leon AC, Olfson M, Portera L, Farber L, Sheehan DV. Assessing psychiatric impairment in primary care with the Sheehan Disability Scale. Int J Psychiatry Med 1997;27:93–105.

2. Posner K, Brown GK, Stanley B, et al. The Columbia–Suicide Severity Rating Scale: initial validity and internal consistency findings from three multisite studies with adolescents and adults. Am J Psychiatry 2011;168:1266–77.

3. E9(R1) statistical principles for clinical trials: addendum: estimands and sensitivity analysis in clinical trials. Guidance for industry (Revision 1). US Department of Health and Human Services, US Food Drug Administration (FDA), Center for Drug Evaluation and Research (CDER), and Center for Biologics Evaluation and Research (CBER), May 2021. (https://www.fda.gov/media/148473/download)

4. Goldstein RB, Smith SM, Chou SP, et al. The epidemiology of DSM-5 posttraumatic stress disorder in the United States: results from the National Epidemiologic Survey on Alcohol and Related Conditions-III. Soc Psychiatry Psychiatr Epidemiol 2016;51:1137–48.

5. Zimmerman M, Benjamin I, Seijas-Rodriguez C. Psychiatric diagnoses among transgender and gender diverse patients compared to cisgender patients. J Clin Psychiatry 2022;83:21m14062.

6. Merrick MT, Ford DC, Ports KA, et al. Vital signs: estimated proportion of adult health problems attributable to adverse childhood experiences and implications for prevention - 25 states, 2015-2017. MMWR Morb Mortal Wkly Rep 2019;68:999–1005.

7. Rosellini AJ, Liu H, Petukhova MV, et al. Recovery from DSM-IV post-traumatic stress disorder in the WHO World Mental Health surveys. Psychol Med 2018;48:437–50.

8. Brooks Holliday S, Dubowitz T, Haas A, Ghosh-Dastidar B, DeSantis A, Troxel WM. The association between discrimination and PTSD in African Americans: exploring the role of gender. Ethn Health 2020;25:717–31.

9. Roberts AL, Gilman SE, Breslau J, Breslau N, Koenen KC. Race/ethnic differences in exposure to traumatic events, development of post-traumatic stress disorder, and treatment-seeking for post-traumatic stress disorder in the United States. Psychol Med 2011;41:71–83.

10. Koenen KC, Ratanatharathorn A, Ng L, et al. Posttraumatic stress disorder in the World Mental Health Surveys. Psychol Med 2017;47:2260–74.

11. Schein J, Houle C, Urganus A, et al. Prevalence of post-traumatic stress disorder in the United States: a systematic literature review. Curr Med Res Opin 2021;37:2151–61.

12. Qassem T, Aly-ElGabry D, Alzarouni A, Abdel-Aziz K, Arnone D. Psychiatric co-morbidities in post-traumatic stress disorder: detailed findings from the adult Psychiatric Morbidity Survey in the English population. Psychiatr Q 2021;92:321–30.

13. de Silva U, Glover N, Katona C. Prevalence of complex post-traumatic stress disorder in refugees and asylum seekers: systematic review. BJPsych Open 2021;7:e194.

14. Holmes SC, Callinan L, Facemire VC, Williams MT, Ciarleglio MM, Smith MV. Material hardship is associated with posttraumatic stress disorder symptoms among low-income Black women. J Trauma Stress 2021;34:905–16.

15. Deck SM, Platt PA. Homelessness is traumatic: abuse, victimization, and trauma histories of homeless men. J Aggress Maltreatment Trauma 2015;24:1022–43.

16. Blais RK, Tirone V, Orlowska D, et al. Self-reported PTSD symptoms and social support in U.S. military service members and veterans: a meta-analysis. Eur J Psychotraumatology 2021;12:1851078.

17. PTSD: National Center for PTSD. Washington, DC: U.S. Department of Veteran Affairs, n.d. (https://www.ptsd.va.gov/understand/common/common_adults.asp)
